# Polar Effects of Transposon Insertion into a Minimal Bacterial Genome

**DOI:** 10.1128/JB.00185-19

**Published:** 2019-09-06

**Authors:** Clyde A. Hutchison, Chuck Merryman, Lijie Sun, Nacyra Assad-Garcia, R. Alexander Richter, Hamilton O. Smith, John I. Glass

**Affiliations:** a Synthetic Biology Group, J. Craig Venter Institute, La Jolla, California, USA; b Synthetic Biology Group, J. Craig Venter Institute, Rockville, Maryland, USA; Princeton University

**Keywords:** minimal cell, polarity, transposon mutagenesis

## Abstract

Global transposon mutagenesis is a valuable tool for identifying genes required for cell viability. Here we present a global analysis of the orientation of viable Tn*5*-Puro^r^ (Tn*5*-puromycin resistance) insertions into the near-minimal bacterial genome of JCVI-syn2.0. Sixteen of the 478 protein-coding genes show a noticeable asymmetry in the orientation of disrupting insertions of Tn*5*-Puro^r^. Ten of these are located in operons, upstream of essential or quasi-essential genes. Inserts transcribed in the same direction as the downstream gene are favored, permitting read-through transcription of the essential or quasi-essential gene. Some of these genes were classified as quasi-essential solely because of polar effects on the expression of downstream genes. Three genes showing asymmetry in Tn*5*-Puro^r^ insertion orientation prefer the orientation that avoids collisions between read-through transcription of Tn*5*-Puro^r^ and transcription of an adjacent gene. One gene (JCVISYN2_0132 [abbreviated here as “_0132”]) shows a strong preference for Tn*5*-Puro^r^ insertions transcribed upstream, away from the downstream nonessential gene _0133. This suggested that expression of _0133 due to read-through from Tn*5*-Puro^r^ is lethal when _0132 function is disrupted by transposon insertion. This led to the identification of genes _0133 and _0132 as a toxin-antitoxin pair. The three remaining genes show read-through transcription of Tn*5*-Puro^r^ directed downstream and away from sizable upstream intergenic regions (199 bp to 363 bp), for unknown reasons. In summary, polar effects of transposon insertion can, in a few cases, affect the classification of genes as essential, quasi-essential, or nonessential and sometimes can give clues to gene function.

**IMPORTANCE** In studies of the minimal genetic requirements for life, we used global transposon mutagenesis to identify genes needed for a minimal bacterial genome. Transposon insertion can disrupt the function of a gene but can also have polar effects on the expression of adjacent genes. In the Tn*5*-Puro^r^ construct used in our studies, read-through transcription from Tn*5*-Puro^r^ can drive expression of downstream genes. This results in a preference for Tn*5*-Puro^r^ insertions transcribed toward a downstream essential or quasi-essential gene within the same operon. Such polar effects can have an impact on the classification of genes as essential, quasi-essential, or nonessential, but this has been observed in only a few cases. Also, polar effects of Tn*5*-Puro^r^ insertion can sometimes give clues to gene function.

## Abstract (precis)

In studies of the minimal genetic requirements for life, we used global transposon mutagenesis to identify genes needed for a minimal bacterial genome. Transposon insertion can disrupt the function of a gene but can also have polar effects on the expression of adjacent genes. In the Tn*5*-Puro^r^ construct used in our studies, read-through transcription from Tn*5*-Puro^r^ can drive expression of downstream genes. This results in a preference for Tn*5*-Puro^r^ insertions transcribed toward a downstream essential or quasi-essential gene within the same operon. Such polar effects can have an impact on the classification of genes as essential, quasi-essential, or nonessential, but this has been observed in only a few cases. Also, polar effects of Tn*5*-Puro^r^ insertion can sometimes give clues to gene function.

## INTRODUCTION

Insertion of a transposable element into a gene interrupts that gene, generally preventing expression of a functional gene product. Consequently, “global transposon mutagenesis” (1) has proven to be a valuable tool for identifying genes required for cell viability. In our investigations of the minimal genetic requirements for life, we have used this method to classify genes as essential (“e”) or quasi-essential (“i”) or nonessential (“n”) (2). Simply put, an essential gene is one for which transposon insertions are lethal, while a nonessential gene can sustain transposon insertions throughout its length with no impairment in growth rate. Transposon insertion into quasi-essential genes results in growth defects. These defects may result in an increased doubling time or may cause cell death after a limited number of divisions.

**FIG 1:**
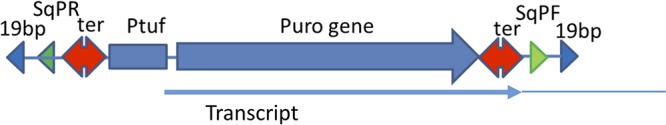
The Tn*5*-Puro^r^ construction used for insertional mutagenesis (2). Transcription of Puro^r^ is driven by the P*tuf* promoter (6). P*tuf*-Puro^r^ is flanked on both sides by a bidirectional transcription terminator (ter), sequencing primers SqPR and SqPF, and the 19-bp terminal repeats from transposon Tn*5*. The weights of the lines at the bottom of the diagram represent the predicted abundance of transcripts initiated at P*tuf* and then attenuated by ter.

However, it has long been known that a transposon insertion into one gene can also have polar effects on the expression of adjacent genes (for examples, see references 3 and 4). In cases where two or more proteins are expressed from a multigene transcript, transposon insertions in one gene can disrupt expression of downstream genes. In our minimal bacterial genome work, we have used a Tn*5*-Puro^r^ (Tn*5*-puromycin resistance) construct (Fig. 1) in which the selectable Puro^r^-encoding gene is driven by the very strong P*tuf* promoter (5, 6). Although we have not experimentally demonstrated read-through, our results presented here strongly support the model that read-through transcription from the Pur^r^ gene can drive transcription of downstream genes, resulting in a selective advantage for Tn*5*-Puro^r^ insertions oriented to transcribe toward “e” or “i” genes located downstream. It should be noted that our minimal cell does not have a gene for termination factor rho and so has only intrinsic (rho-independent) termination. It has been established that intrinsic termination permits significant read-through in other species. For example, an analysis of 13 rho-independent terminators in Escherichia coli showed that the efficiency of termination *in vitro* ranged from only 2% to 90% (7).

Here we present a global analysis of the orientation of Tn*5*-Puro^r^ insertions in our near-minimal cell, JCVI-syn2.0. This analysis gives clues to polar effects within multigene transcription units that can affect the classification of genes as “e,” “i,” or “n” and can give clues to gene function in some cases.

## RESULTS

### Some genes show a directional bias for Tn*5*-Puro^r^ insertion events.

We have analyzed transposon insertion data to identify insertions in the forward and reverse orientations in the near-minimal genome of JCVI-syn2.0 (2). This data set was chosen for analysis because of its high density of transposon insertion sites. This data set contains Tn*5*-Puro^r^ insertions that map to 15,852 unique sites in the syn2.0 genome. About half of the 576,028-bp genome is composed of essential genes and therefore cannot tolerate Tn*5*-Puro^r^ insertions. So the density of insertions averaged over the quasi-essential genes, intergenic regions, and the remaining nonessential genes is approximately 1 per 20 bp. The numbers of insertions in the two directions differ significantly in only a small number of cases. Table 1 lists 16 of 478 protein-coding genes in JCVI-syn2.0 that showed a noticeable skew in the directionality of viable transposon insertions. We define a directional skew parameter (*S*) that ranges from 0, where the numbers of forward (#*F*) and reverse (#*R*) insertions are equal, to 1, where there are insertions in a gene and all are in one direction. *S* is calculated as S=ABS[(#F−#R)/(#F+#R)], where ABS indicates an absolute value. We arbitrarily define *S* = 0 when a gene has no insertions and the equation above gives a divide-by-0 error. The values of *S* are listed in Table 1 for each of the 16 genes showing a noticeable skew (see Table 1, footnote *a*, for details).

**TABLE 1:** Genes of JCVI-syn2.0 that show a skew in the orientation of transposon insertions^*a*^

Beg	End	Direction	Preferred insertion orientation	RGD fragment	Length (aa)	Locus tag	Gene HSW 150126	P1F	P1R	P1F:R	P2F	P2R	P2F:R	P6F	P6R	P6F:R	Likely reason	Flanking gene affected	New context in syn2.0?	Current annotation	Functional classification	System
2675	3217	f	f	1	180	_0003	i	5	0	1	6	3	0.33	1	0	0.99	Upstream train wreck avoided	(_0002) DNA Pol III beta subunit	No	RNase MS	Equivalog	Ribosome biogenesis
55909	56499	r	r	1	196	_0046	n	0	12	1	3	13	0.62	0	4	1	Downstream read-through needed	(_0045) thymidylate kinase	No	Recombination protein	Equivalog	DNA repair
63727	64701	f	f	1	324	_0063	n	22	7	0.52	53	12	0.63	22	2	0.83	Downstream read-through needed	(_0064) lysyl tRNA synthetase	No	Uncharacterized tRNA dihydrouridine synthase	Generic	tRNA modification
74697	75536	r	r	1	279	_0077	n	1	9	0.8	3	27	0.8	0	4	1	Downstream read-through needed	(_0076) asparaginal tRNA synthetase	No	Low-specificity hydrolase	Putative	Unclear
77752	77973	r	r	1	73	_0080	n	0	6	1	2	10	0.67	0	2	1	?	?	No	Uncharacterized protein	Unknown	Unclear
87708	88820	r	r	2	370	_0108	i	3	15	0.67	2	12	0.71	0	2	1	Downstream read-through needed	(_0107) *nusB* transcription antitermination	No	Lipoprotein, putative	Generic	Lipoprotein
91384	192304	r	f	2	306	_0114	i	4	1	0.6	6	2	0.5	0	0	0	Downstream train wreck avoided	(_0113) glycosyl transferase, group 2	No	Glycosyltransferase, group 2 family protein	Putative	Lipid salvage and biogenesis
101419	102480	f	r	2	353	_0132	i	0	3	1	0	7	1	0	0	0	Downstream read-through lethal	(_0133) toxin-peptidase, S8/S53 family	No	Toxin-antitoxin AAA ATPase	Probable	Toxin-antitoxin
125069	125563	r	r	2	164	_0164	i	0	6	1	1	4	0.6	0	0	0	Downstream read-through needed	(_0163) alanyl tRNA synthetase	No	Hypothetical protein	Unknown	Unclear
204874	205368	r	r	3	164	_0301	i	2	6	0.5	0	1	0.99	0	0	0	Downstream read-through needed	(_0300) *nusA* transcription termination/antitermination	No	*rimP*	Putative	Ribosome biogenesis
294566	297532	r	r	4	988	_0415	i	1	6	0.71	1	3	0.5	0	0	0	?	?	No	Chromosome segregation protein SMC	Equivalog	Chromosome segregation
468223	469179	r	r	7	318	_0697	n	4	11	0.47	3	5	0.25	0	8	1	Downstream read-through needed	(_0696) RDD family protein	Yes (upstream)	Glycosyltransferase, group 2 family protein	Generic	Unclear
477490	478326	f	f	7	278	_0728	i	15	5	0.5	6	1	0.71	0	0	0	Downstream read-through needed	(_0729) phosphoglycerate mutase	No	HAD hydrolase, family IIB	Generic	Unclear
483396	484049	f	f	7	217	_0747	i	9	1	0.8	0	2	1	0	0	0	?	?	Yes (up- and downstream)	*punA*	Probable	Nucleotide salvage
506742	507185	r	r	8	147	_0800	i	1	9	0.8	1	0	0.99	0	0	0	Downstream read-through needed	(_0799) *glyA*, serine hydroxymethyltransferase	Yes (upstream)	*rpiB*: ribose 5-phosphate isomerase B	Equivalog	Metabolic process
514997	516172	r	r	8	391	_0805	i	2	18	0.8	0	6	1	0	2	1	Downstream read-through needed	(_0804) RNA Pol, beta subunit	No	Transcription factor	Generic	Regulation

a Data correspond to the indicated 16 genes that show a skew in the orientation of viable Tn*5*-Puro^r^ insertions. The coordinates of each gene on the JCVI-syn2.0 genome (GenBank accession no. CP014992.1) are listed in the “Beg” (beginning) and “End” columns. Columns P1F, P2F, and P6F list the numbers of different insertion sites for Tn*5*-Puro^r^ in a particular gene in the “forward orientation” observed for passages 1, 2, and 6. The analogous numbers of insertion sites in the “reverse orientation” are shown in columns P1R, P2R, and P6R. Skew values were calculated for each gene at generations 1, 2, and 6 as described in the text, and data are listed as PnF:R (where “n” represents the passage number). For example, the skew value for gene _0046 in passage 2 is P2F:R = ABS (3 − 14)/16 = 0.62. A likely reason for the observed skew is listed where the data suggest one (see column “Likely reason”). The flanking gene whose functionality likely depends on the orientation of the Tn*5*-Puro^r^ insertion is indicated in the “Flanking gene affected” column. If the context of the gene in JCVI-syn2.0 is different from that in JCVI-syn1.0, due to removal of flanking genes, then this is indicated. Information concerning the annotation for each listed gene is shown in the last three columns, “Current annotation,” “Functional classification,” and “System” (updated from a previously described annotation [2]). aa, amino acids; Equivalog, gene (or corresponding encoded protein) that is known to have been conserved in function since the last common ancestral sequence; Pol III, polymerase III; RGD, Arg-Gly-Asp.

When there is a directional skew of insertion events in a gene, we assume that this results from an effect of these insertions on the expression of adjacent genes. We have been able to explain most of the observed skewing as the result of read-through transcription of adjacent genes. We describe several specific examples below.

### In some cases, read-through transcription of Tn*5*-Puro^r^ inserted in upstream genes of an operon allows normal function of essential downstream genes, but in other cases it does not.

**FIG 2:**
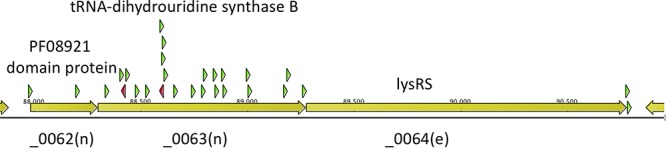
In some cases, read-through from Tn*5*-Puro^r^ is sufficient to drive a downstream essential gene. Results are shown for passage 6 following harvesting a pool of puromycin-resistant transformants. Green triangles represent insertions in the forward orientation, and red triangles indicate the reverse orientation.

An example of a three-gene transcription unit (genes JCVISYN2_0062 [“_0062”] to _0064) for which Tn*5*-Puro^r^ insertion in an upstream gene (_0062 or _0063) allows normal function of the essential downstream gene for lysyl-tRNA synthetase (*lysRS*, gene _0064) is shown (Fig. 2). This is evidenced by the recovery of insertions into the upstream genes following 6 serial passages in liquid medium. There is a strong preference for upstream Tn*5*-Puro^r^ insertions oriented to transcribe toward *lysRS*, indicating that read-through transcription from Tn*5*-Puro^r^ is required for LysRS expression.

**FIG 3:**
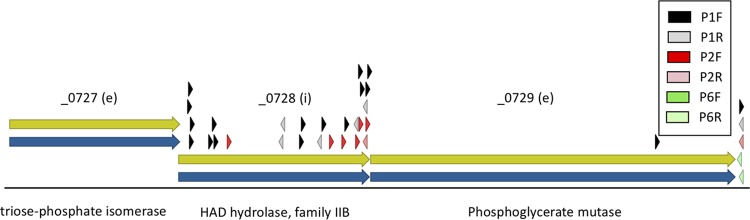
Gene _0728 appears quasi-essential (i), apparently due to a polar effect on expression of the downstream essential (e) gene _0729. The notation “(e)” indicates essential genes, and “(i)” indicates a quasi-essential gene. Arrowheads indicate the positions of Tn*5*-Puro^r^ insertions. In the color code shown, “P1” represents passage 1, “P2” indicates passage 2, and “P6” indicates passage 6. Right-pointing arrowheads indicate insertions in forward orientation, while left-pointing arrowheads indicate reverse orientation.

However, it is clear that read-through from Tn*5*-Puro^r^ is not always sufficient to allow normal function of downstream genes. We show a three-gene transcription unit consisting of genes _0727 to _0729 (Fig. 3). The first and third genes are essential glycolytic enzymes, while the middle gene, HAD hydrolase _0728, appears to be quasi-essential as judged by our usual criteria. Tn*5*-Puro^r^ insertions were recovered in _0728 but with a strong bias for the forward orientation, which would produce read-through transcripts driving expression of the essential phosphoglycerate mutase gene that lies downstream (_0729). Gene _0728 was originally classified as quasi-essential because insertions were observed for passage 1 (P1) and P2 but not for P6, indicating a growth defect. However, the directional bias of the insertions suggested that the growth defect could result from reduced expression of downstream gene _0729 rather than directly from the loss of _0728 function.

In other experiments aimed at studying the possible role of HAD hydrolases in metabolite damage control, we made a single-gene knockout of _0728 in the background of JCVI-syn3A. This knockout has no detectable effect on growth rate. Taken together, these results make a convincing argument that function of gene _0728 is not needed for normal growth, and it was classified as quasi-essential because of effects on gene _0729 expression.

Among the 16 genes in Table 1 with directionally skewed insertions, 9 appear to be associated with the need for downstream read-through to drive an essential (“e”) gene, as in the two examples discussed above. Of these 9, we had classified 4 as nonessential (“n”) (genes _0046, _0063, _0077, and _0697) and 5 as quasi-essential (“i”) (genes _0108, _0164, _0301, _0728, and _0805) based on total numbers of insertions, without considering the directions of the insertions. One gene (_0800, an “i” gene) has directionally skewed insertions which appear to result from the need for read-through transcription of downstream quasi-essential “i” gene _0799. Any of these six genes initially classified as “i” may actually be completely dispensable, as in the case of _0728, and may have no significant role in cell growth.

The other 6 genes listed in Table 1 are not explainable in terms of the need for transcriptional read-through to downstream genes. We consider these genes below.

### Evidence for a toxin-antitoxin gene pair.

**FIG 4:**
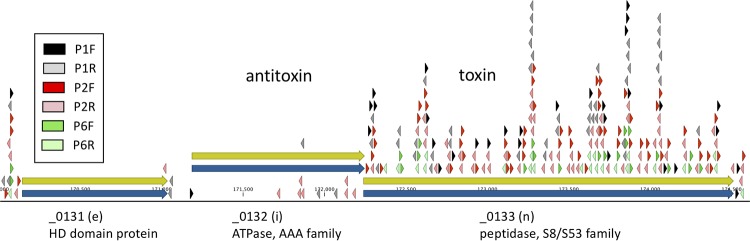
Gene _0132 shows an asymmetry in Tn*5*-Puro^r^ insertions that can be explained by a toxin-antitoxin system where _0133 is the toxin and _0132 is the antitoxin. The notation “(e)” indicates an essential gene, “(n)” indicates a nonessential gene, and “(i)” indicates a gene originally categorized as quasi-essential. Arrowheads indicate the positions of Tn*5*-Puro^r^ insertions. In the color code shown, “P1” represents passage 1, “P2” indicates passage 2, and “P6” indicates passage 6. Right-pointing arrowheads indicate insertions in forward orientation, while left-pointing arrows indicate reverse orientation.

Gene _0132 is upstream of _0133, and the two genes appear to be cotranscribed into a single message (Fig. 4). _0132 is classified as quasi-essential (i), and gene _0133 is nonessential. Transpositions into _0132 are strongly biased in favor of transcription upstream, away from _0133. These findings are consistent with a model in which expression of _0133 in the absence of _0132 expression is lethal. This relationship suggested the possibility that these genes form a toxin-antitoxin (TA) pair. Both genes were classified as “generic” in our annotation of JCVI-syn3.0, with the antitoxin (_0132) annotated as an ATPase (AAA family) and the toxin (_0133) as a peptidase (S8/S53 family). A search of the literature reveals a very similar plasmid-borne toxin-antitoxin system in Agrobacterium tumefaciens, which also consists of a serine protease and an AAA-ATPase (8). These findings support the hypothesis that genes _0133 and _0132 (_0133/_0132) constitute a TA pair. TA systems are commonplace bicistronic bacterial genes that frequently regulate growth of the host. While the targets of many systems are unknown, bacteriostatic systems involving numerous aspects of translation, replication, ATP generation, cell wall synthesis, cell division, and cell membranes have been described previously. To confirm our hypothesis, we have shown that simultaneous knockouts of the toxin-antitoxin pairs of genes give a viable cell, whereas a knockout of the antitoxin gene alone is lethal. The gene pair _0132/_0133 was knocked out from the genome of strain syn3A genome (9) (GenBank accession no. CP016816.2). The genome was propagated in Saccharomyces cerevisiae (yeast), and the knockout was made using clustered regularly interspaced short palindromic repeats-Cas9 (CRISPR/Cas9). Separately, gene _0132 was knocked out from the syn3A genome by the same method. Genomes from yeast carrying syn3A with the _0132/_0133 knockout gave a viable cell, and Sanger sequencing of a PCR product spanning the deletion junction confirmed that both genes were knocked out. Genome transplantation from yeast carrying syn3A with the _0132 knockout did not produce any viable transplants. Because transplantation is a difficult procedure, a negative result is not always conclusive. For this reason, gene _0132 flanked by two *loxP* sites was reinserted into the syn3A genome propagated in yeast and carrying the _0132 knockout. Transplantation yielded a viable bacterial cell with a single copy of gene _0132 flanked by two *loxP* sites. This cell was then transformed with plasmid Pmod2loxpurolox-cresp, which carries the Puro^r^ gene flanked by two *loxP* sites, and transformants were selected for puromycin (Puro) resistance to select cells in which gene _0132 was replaced by the Puro^r^ gene. Only a few puromycin-resistant colonies were found, and these carried a mutation in gene _0133, presumably inactivating the toxic effect of the _0133 gene product. A control experiment performed with the nonessential gene _0728 gave thousands of colonies. In other experiments directed at further reducing the minimal cell genome, _0133 was knocked out while leaving _0132 intact, with no noticeable effect on growth. This result is also consistent with our model. Taken together, these experiments demonstrated that knockout of _0132 from a syn3A genome while leaving an intact wild-type gene (_0133) was lethal, providing strong evidence that the _0132/_0133 gene pair constitutes an antitoxin/toxin pair.

### Asymmetry in directions of transposon insertions sometimes results in avoidance of a transcriptional “train wreck” with an adjacent gene.

**FIG 5:**
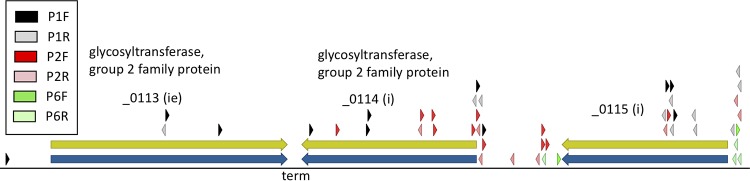
Gene _0114 shows an asymmetry in Tn*5*-Puro^r^ insertions that may result from the potential for a “train wreck” with the _0113 transcript. The notation “ie” indicates a gene on the borderline between essentiality and quasi-essentiality, and “i” indicates quasi-essential genes. Arrowheads indicate the positions of Tn*5*-Puro^r^ insertions. In the color code shown, “P1” represents passage 1, “P2” indicates passage 2, and “P6” indicates passage 6. Right-pointing arrowheads indicate insertions in forward orientation, while left-pointing arrowheads indicate reverse orientation. “term” indicates the location of a bidirectional stem-loop transcriptional terminator.

In Fig. 5, we show an example of a skew in the orientation of Tn*5*-Puro^r^ insertions that resulted in avoidance of a transcriptional conflict with an adjacent gene. Such a conflict might involve RNA polymerase collisions or might result from production of an antisense transcript that interferes with translation of the adjacent gene. Genes _0113 and _0114 are both annotated as encoding glycosyltransferase (group 2 family proteins). They are believed to be involved in lipid salvage and biogenesis. The two genes are transcribed in opposite directions and toward a bidirectional transcription terminator in the sequence separating them. This terminator serves to transcriptionally isolate the two genes, presumably preventing a transcriptional conflict. Gene _0113 is classified as “ie” because it contains only a very low number of insertions and only at an early passage step (P1). Gene _0114 is classified as “i” and shows a marked preference for insertions directed away from gene _0113. We hypothesize that this orientation is preferred because it avoids a transcriptional conflict with gene _0113. Such a train wreck could be produced by read-through of the intergenic terminator by transcripts from the very strong P*tuf* promoter driving Tn*5*-Puro^r^.

Another gene listed in Table 1 (locus tag _0003) shows an asymmetry in insertions that may have resulted from selection against a “train wreck” with transcription of the upstream gene (see Fig. S1 in the supplemental material).

### In some cases, we see no obvious explanation for the asymmetry in the orientation of transposon insertions.

The three remaining genes listed in Table 1 (_0080, _0415, and _0747) show a preference for Tn*5*-Puro^r^ insertions that are transcribed in the same direction as the gene. In each of these three cases, there is a sizable intergenic region upstream of the gene (199 bp, 305 bp, and 363 bp, respectively). It seems possible that read-through transcription from Tn*5*-Puro^r^ into these intergenic regions is selected against, leading to the observed bias in insertion orientations (Fig. S2, S3, and S4).

## DISCUSSION

Insertion of a transposable element into the central region of a gene almost always inactivates the function of the gene, leading to cell death if function of the gene is required for viability. Global transposon mutagenesis has been used to identify the set of nonessential genes, and the sets of essential and quasi-essential genes have been estimated by process of elimination. This paper considers the complications introduced into the analysis of global transposon mutagenesis data by the existence of multigene transcription units (operons). Insertion of the Tn*5*-Puro^r^ sequence into one gene of an operon could potentially block expression of downstream genes in that operon. We have detected such effects by analyzing the orientation of Tn*5*-Puro^r^ sequence insertions with respect to the direction of transcription within the operon. When read-through transcription of a downstream gene is required for normal growth following insertional disruption of a gene, a preference for Tn*5*-Puro^r^ insertions transcribed in the same direction as the operon is then expected. We have defined a directional skew parameter for the Tn*5*-Puro^r^ insertions within a gene and calculated it for the 478 protein-coding genes of JCVI-syn2.0. This paper presents an analysis of 16 genes of JCVI-syn2.0 that show evidence of a significant skew in the orientation of Tn*5*-Puro^r^ insertions.

Our analysis suggests that read-through transcription from the Tn*5*-Puro^r^ gene is sufficient, in some cases, to drive transcription of downstream essential genes, thereby allowing normal growth (see results concerning the transcription unit consisting of gene _0062 to gene _0064 in Fig. 2). In other cases, read-through transcription appears to allow only a reduced level of downstream gene function (see results concerning the transcription unit consisting of gene _0727 to gene _0729 in Fig. 3). In this case, it appears that _0728 was classified as “i” (quasi-essential) because of a polar effect on the expression of _0729 when the gene was disrupted by Tn*5*-Puro^r^ transposition. Actual knockout of _0728 did not cause a noticeable growth defect.

In summary, about 3% (16 of 478) of the protein-coding genes in JCVI-syn2.0 show a noticeable asymmetry in the orientation of disrupting insertions of Tn*5*-Puro^r^ sequence. This is a small fraction of the genes, indicating that polar effects have a relatively minor impact on the classification of genes as essential (“e”), quasi-essential (“i”), or nonessential (“n”). The majority of these genes (10 of 16) are located in operons, upstream of essential or quasi-essential genes, and insertions transcribed in the same direction as the downstream gene are favored. The skew in Tn*5*-Puro^r^ orientation can be explained as a requirement for transcriptional read-through of the Tn*5*-Puro^r^ gene, in order to drive downstream genes. Of these 10 genes, 4 were classified as nonessential (“n”) and 6 as quasi-essential (“i”). One of these genes (_0728) showed no noticeable growth defect when excised from the genome, suggesting that the other 5 may also be candidates for removal.

It seems possible that some genes may be classified as “e” (essential) genes because Tn*5*-Puro^r^ disruption impairs function of a downstream “e” gene rather than because of a direct effect of loss of the disrupted gene’s function. However, so far, we have no evidence representing a specific example of this possibility.

The skew in orientation of Tn*5*-Puro^r^ insertions in the remaining 6 genes cannot be explained in terms of effects of gene disruption on the transcription of downstream genes, and a consideration of these follows.

Perhaps the most interesting result of this study is the finding that genes _0133 and _0132 appear to form an antitoxin/toxin pair (a TA pair). The discovery was made because insertions of Tn*5*-Puro^r^ sequence into the antitoxin gene are skewed to result in transcription upstream, away from the toxin gene (Fig. 4). This indicates that expression of gene _0133 (toxin) is lethal when gene _0132 (antitoxin) is disrupted. It seems likely that a similar skew in transposon orientation would occur in other TA systems with the same transcriptional architecture—regardless of the enzymatic activities involved. Directional bias might therefore be used to uncover novel TA systems. This is an intriguing possibility, in light of the recent appreciation of the role that TA systems sometimes play in biofilms and in pathogen persistence (for a review, see reference 10). This particular TA system consists of the antitoxin (_0132) annotated as an ATPase (AAA family) and the toxin (_0133) annotated as a peptidase (S8/S53 family). This is very similar to a TA pair previously described in Agrobacterium tumefaciens (8). Although the identification of this TA pair is quite clear, we do not know the mechanism of action of the peptidase toxin. The toxic effect could result from specific cleavage of some target protein or from a general proteolytic activity. The antitoxin may inhibit the toxin by binding to the toxin to form an inactive complex. Our results led us to make the following predictions, which have been borne out by experiment. (i) TA gene pair _0132 and _0133 can be excised from the genome without impairing growth. (ii) Excision of _0132 (antitoxin) alone is lethal. (iii) When gene _0133 (toxin) is excised while leaving gene _0132 (antitoxin) in place, there is no noticeable impairment of growth, as would be expected according to our model. To our knowledge, this is the first experimental evidence for the presence of a TA pair in a mycoplasma. It is worth noting that a homologous pair of genes can be readily identified in other species of mycoplasma by means of BLAST searches. For example, in Mycoplasma gallisepticum, a highly similar pair of genes is found, again annotated as an “AAA family ATPase” gene and an “S8 family peptidase” gene, but with no indication that they form a TA pair. Since we made our observations, a bioinformatic approach has identified some putative TA systems in certain members of the *Mycoplasm*a genus (11).

Two of the remaining genes show asymmetries in Tn*5*-Puro^r^ sequence insertions oriented to avoid collisions between the read-through transcription of Tn*5*-Puro^r^ and the transcription of the upstream gene, thereby avoiding a “transcriptional train wreck” (genes _0003 and _0114).

The three remaining genes listed in Table 1 (genes _0080, _0415, and _0747) show a predominance of insertions oriented to transcribe in the same direction as the gene, but read-through transcription does not appear to be a factor (see Fig. S2, S3, and S4 in the supplemental material). The only common feature that we have noted is that read-through transcription of Tn*5*-Puro^r^ is preferentially directed downstream of the gene and away from a sizable upstream intergenic region (199 to 363 bp). It seems possible that these intergenic regions specify some unannotated essential functions and that read-through transcription of Tn*5*-Puro^r^ into these intergenic sequences interferes with expression of those functions. For example, unannotated small RNAs or riboswitches might reside in the intergenic region.

Overall, the analysis of directional bias in the orientation of Tn*5*-Puro^r^ insertions has revealed a small number of genes in JCVI-syn2.0, previously classified as quasi-essential, that may be removable without causing impairment of growth. Analysis of directional bias can sometimes give clues to gene function, as in the case of a TA (toxin/antitoxin) gene pair. Several unexplained examples of genes with a skewed distribution of Tn*5*-Puro^r^ insertions require further explanation. Thus, analysis of the orientation of insertions in global transposon mutagenesis can result in refinements in classifying gene essentiality and in some cases can give clues to gene function.

## MATERIALS AND METHODS

This paper presents a reanalysis and interpretation of previously published data as described below.

### JCVI-syn2.0 gene designations.

The near-minimal cell JCVI-syn2.0 has been previously described (2) (GenBank accession no. CP014992.1 [576,026 bp]). For brevity, we usually refer to its genes using their GenBank locus tags but omitting the leading “JCVISYN2.” Thus, gene JCVISYN2_0132 is referred to here as “gene _0132” or simply “_0132.” The near-minimal JCVI-syn2.0 has a subset of the genes of JCVI-syn1.0 (GenBank accession no. CP002027 [1,078,809 bp]). The numerical part of the locus tag for each gene of JCVI-syn2.0 is the same as for the identical gene found in JCVI-syn1.0. Thus, for example, gene JCVISYN2_0132 in JCVI-syn2.0 has the same sequence as gene MMSYN1_0132 in JCVI-syn1.0. We indicate whether a gene is essential (e), quasi-essential (i), or nonessential (n) by including its classification in parentheses following the locus tag designation. Thus, “_0132 (i)” indicates that gene _0132 is quasi-essential.

### Determination of gene essentiality using Tn*5*-Puro^r^ transposition.

We used the Tn*5*-Puro^r^ construct diagrammed in Fig. 1, rather than a naturally occurring transposon, for global transposon mutagenesis of our mycoplasma-based synthetic cell JCVI-syn1.0 (12). The studies performed as previously described (2) allowed us to identify which genes to include in our designs for the reduced genomes of JCVI-syn2.0 (576,026 bp; GenBank accession no. CP014992.1) and syn3.0 (531,490 bp; GenBank accession no. CP014940.1). In these experiments, Tn*5*-Puro^r^ DNA was bound to Tn*5* transposase *in vitro* and then introduced into a cell population by polyethylene glycol (PEG)-mediated transformation. Populations of puromycin-resistant colonies were pooled after they were harvested from solid media, followed by 4 to 6 serial passages of these populations in liquid culture. There are approximately 10 cell doublings per passage. This procedure strongly selects against Tn*5*-Puro^r^ insertions that result in lethality or in an increase in cell doubling time. Junctions between Tn*5*-Puro^r^ and the genome were amplified by an inverse PCR strategy and were subjected to deep sequencing (2). The insertion points were then mapped onto the genome, and the genes were classified as “e,” “i,” or “n.” Nonessential (n) genes show transposon insertions in the original population recovered from solid media (P0) and also after extended passage (up to P6; for example, see gene _0133 data in Fig. 4). Quasi-essential (i) genes have transposon insertions at P0 but with a reduced number at higher passage numbers (for example, see gene _0115 data in Fig. 5). Essential (e) genes contain no transposon insertions or, in some cases, a very few located near the 3′ end of the gene (for examples, see data corresponding to genes _0727 and _0729 in Fig. 3).

### Orientation-dependent consequences of T*n5*-Puro^r^ insertion events.

**FIG 6:**
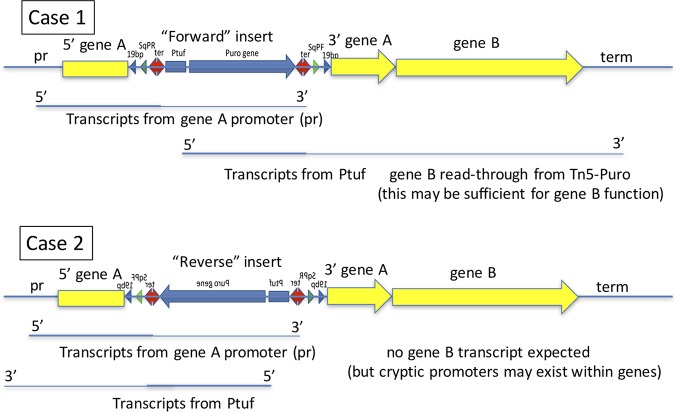
Consequences of “forward” and “reverse” insertion of Tn*5*-Puro^r^. The Tn*5*-Puro^r^ sequence is shown inserted in gene A of hypothetical two-gene transcription unit AB in both orientations. Weights of lines represent relative transcript abundances, which change as a consequence of attenuation by ter.

When the Tn*5*-Puro sequence (Fig. 1) is inserted into the cellular genome, transcription from the P*tuf* promoter (6) is attenuated by the downstream terminator but reads through at a reduced level into the genomic sequence in which the element is inserted. A hypothetical situation that illustrates how cell viability could depend on the orientation of Tn*5*-Puro^r^ insertions at a particular site is shown in Fig. 6. In this example, a completely dispensable gene (gene A) is cotranscribed with a downstream gene (gene B) that is required for cell viability. Consider the consequences of insertion of the Tn*5*-Puro^r^ sequence in each of the two orientations, at the same site within gene A. In case 1, both the Tn*5*-Puro^r^ gene and genes A and B are transcribed in the “forward” direction (5′ to 3′ on the genome sequence as written in its conventional orientation). In case 2, transcription of Tn*5*-Puro^r^ is in the “reverse” direction, opposite the direction of transcription of genes A and B. In both orientations, we expect Tn*5*-Puro^r^ insertion within gene A to block transcripts initiated by the gene A promoter “pr” from proceeding through gene B. In case 1 (“forward insert”), transcription from the P*tuf* promoter is attenuated but proceeds through gene B at a reduced level. This level of transcription may be sufficient to allow normal levels of the gene B product to be expressed. In case 2 (“reverse insert”), we expect to see no transcript of gene B. Since gene B function is required for viability and A is dispensable, we expect that “reverse” insertions in gene A would be lethal because they block expression of downstream gene B. However, “forward” insertions may be viable, due to read-through from the P*tuf* promoter. Consequently, we expect that any Tn*5*-Puro^r^ insertions within gene A in viable cells would be predominantly in the “forward” orientation.

### Determination of the orientation of Tn*5*-Puro^r^ insertions.

**FIG 7:**
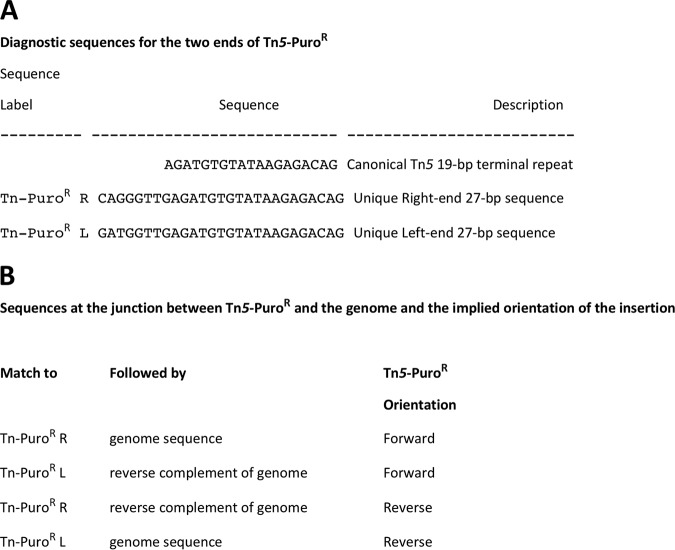
Unique 27-bp sequences at the ends of Tn*5*-Puro^r^ flank the junction with genomic sequence. The unique sequences from the right and left ends of Tn*5*-Puro^r^ are shown (see the supplemental material for reference 2 for the complete sequence). Sequences are all shown in the 5′ to 3′ orientation such that the Tn*5*-Puro^r^ L unique 27-bp sequence is the reverse complement of the Tn*5*-Puro^r^ sequence as conventionally written.

In our published analyses of global transposon mutagenesis data, we have not previously considered orientation of the transposon insertions. However, our sequence data contain information that allows determination of insertion orientation. The 19-bp sequences at the two ends of the Tn*5*-Puro^r^ insertion are identical, but sequences of 25 bp or longer become unique. The unique 27-bp right-end and left-end sequences for Tn*5*-Puro^r^ are shown in Fig. 7A. A search for unique left-end and right-end sequences followed by either a match to the genome (written in its standard orientation) or a match to the reverse complement of the genome resulted in identification of 4 cases as listed in Fig. 7B. Classification of the Tn*5*-Puro^r^ junctions with the genome into these 4 cases allows determination of the orientation (forward or reverse) of each insertion event.

A simple Python script was used to locate insertions and assign their orientations. Fastq files were first searched for sequences with a perfect match to the last 25 nucleotides (nt) at the primer SqPF end of the Tn*5*-Puro^r^ construct (Fig. 1). For cases in which an additional 25 nt of putative genomic sequence were present in the sequencing read, the candidate sequence was compared to a hash of all possible substrings (of length 25 nt) from the top and bottom strands of JCVI-syn2.0. Perfect matches to the genome were required, and only insertions at unique substrings were used. A forward or reverse orientation was then assigned based on whether the match was from the top strand or the bottom strand of the genome. Multiple insertions at the same site and in the same orientation were assumed to represent a single transposon insertion event—the library had undergone multiple rounds of amplification, and it was impossible to determine if multiple insertions represented independent events or biased amplification. The Python code is included in the supplemental material.

### Gene knockouts produced by using CRISPR/Cas9.

Gene _0132, genes _0132 and _0133, and gene _0728 were each knocked out from the syn3A genome (9) carried in yeast strain VL6**-**48N**-**Cas9 (a gift from Daniel G. Gibson). Strain VL6**-**48N**-**Cas9 carries the *cas9* gene in the yeast genome and expresses CAS9 constitutively, as described by Kannan et al. (13). The syn3A genome was introduced into VL6-48N-Cas9 by fusion of syn3A with spheroplasts of VL6-48N-Cas9. To obtain a knockout-appropriate guide RNA(s), a donor DNA and a plasmid carrying a selectable marker (*trp1* or *ura3*) were simultaneously introduced into the yeast by electroporation (see the supplemental material for more details). Colonies grown under selective conditions for the plasmid marker were screened by PCR to identify those carrying the desired gene knockouts. Genome transplantation from yeast into M. capricolum was then performed to recover syn3A as a bacterial strain with the desired gene knockout. See reference 13 for detailed methods.

## Supplementary Material

Supplemental file 1
