# Supplementary material for: Polar Effects of Transposon Insertion into a Minimal Bacterial Genome
Source: J Bacteriol. 2019 Sep 6;201(19):e00185-19. doi: 10.1128/JB.00185-19 (PMC6755753; doi:10.1128/JB.00185-19)
Supplement: Supplemental file 1 [file JB.00185-19-s0001.pdf]

Polar effects of transposon insertion into a minimal bacterial genome

Clyde A. Hutchison III[a#], Chuck Merryman[a], Lijie Sun[a], Nacyra Assad-Garcia[b], R. Alexander Richter[a], Hamilton O. Smith[a], John I. Glass[a]

[a] Synthetic Biology Group, J. Craig Venter Institute, La Jolla, California 92037

[b] Synthetic Biology Group, J. Craig Venter Institute, Rockville, Maryland 20850

[#] Address correspondence to Clyde A. Hutchison III, chutchis@jcvi.org

Supplementary Materials

Supplementary figures S1-S12 show Tn*5*-Puro^R insertions in 12 genes that exhibit a skew in insert orientation, and are not shown in the body of the paper.

<u>The following conventions apply to all figures S1-S12</u>. The degree of essentiality of each gene is indicated in parentheses following the abbreviated locus tag:

e indicates an essential gene

i indicates a quasi-essential gene

n indicates a non-essential gene

ie indicates a gene on the borderline between quasi-essential and essential

in indicates a gene on the borderline between quasi-essential and non-essential

So, for example, _0002 (e) indicates that gene _0002 is an essential gene.

The insertion sites for Tn*5*-Puro^R are indicated by triangular arrowheads. Right-pointing triangles indicate insertions in the forward orientation, and left-pointing triangles indicate insertions in the reverse orientation. The arrowheads are colored as follows:

P1F	Passage 1, forward orientation
P1R	Passage 1, reverse orientation
P2F	Passage 2, forward orientation
P2R	Passage 2, reverse orientation
P6F	Passage 6, forward orientation
P6R	Passage 6, reverse orientation

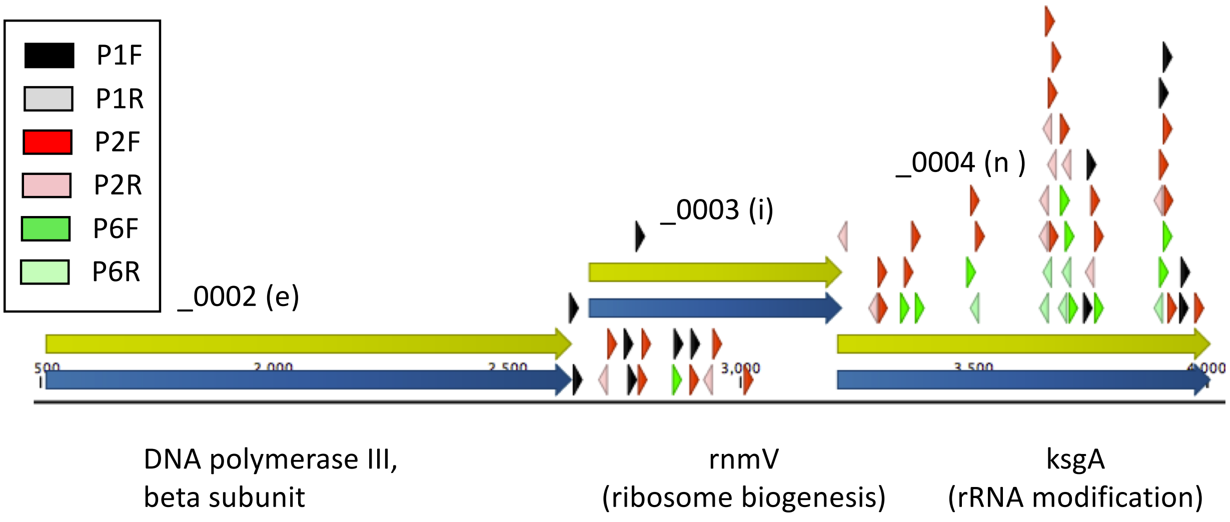

Fig. S1 **Gene _0003 shows an asymmetry in Tn*5*-Puro^R insertions that may result from the potential for a "train wreck" with the _0002 transcript.** Gene _0002 is an essential gene required for DNA replication. Read-through transcription of Tn*5*-Puro^R inserts in _0003 are not needed for _0004 expression because it is a non-essential (n) gene.

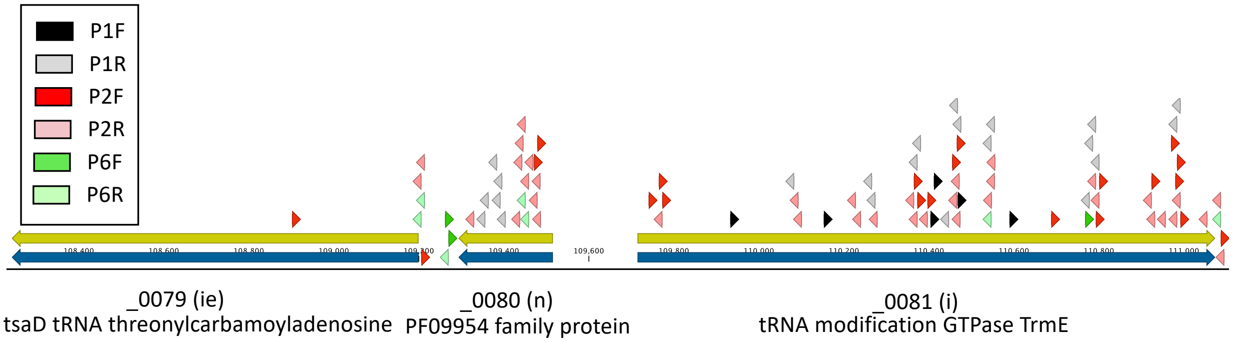

Fig. S2 **Gene _0080 shows an asymmetry in Tn*5*-Puro^R insertions for no obvious reason.**

The absence of Tn*5*-Puro^R insertions in gene _0080 that are oriented to produce read-through transcription into the upstream intergenic region suggests that such read-through is selected against. Also, this intergenic region does not have any Tn*5*-Puro^R insertions. Taken together these findings suggest that the intergenic region (_0080-_0081) specifies some necessary function that can be blocked either directly by Tn*5*-Puro^R insertion, or by read-through transcription from Tn*5*-Puro^R. This intergenic region apparently must contain promoters in both directions for transcription of genes _0080 and _0081. It seems possible that transcriptional read-through from a Tn*5*-Puro^R insert in gene _0080 could interfere with transcription from the normal promoter for _0081. This could result in a lower level of expression of gene _0081, a strongly quasi-essential gene, producing selection against such events. Alternatively, it seems possible that some unannotated essential function resides in the intergenic region between genes _0080 and _0081 and that read-through transcription from Tn*5*-Puro^R interferes with its expression.

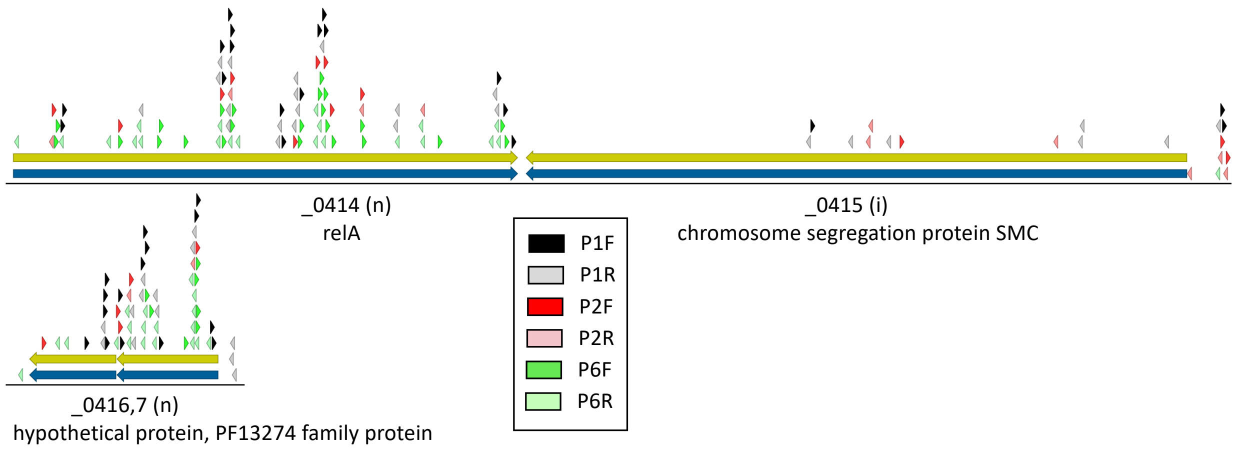

Fig. S3. **Gene _0415 shows an asymmetry in Tn*5*-Puro^R insertions but the reason is not obvious.**

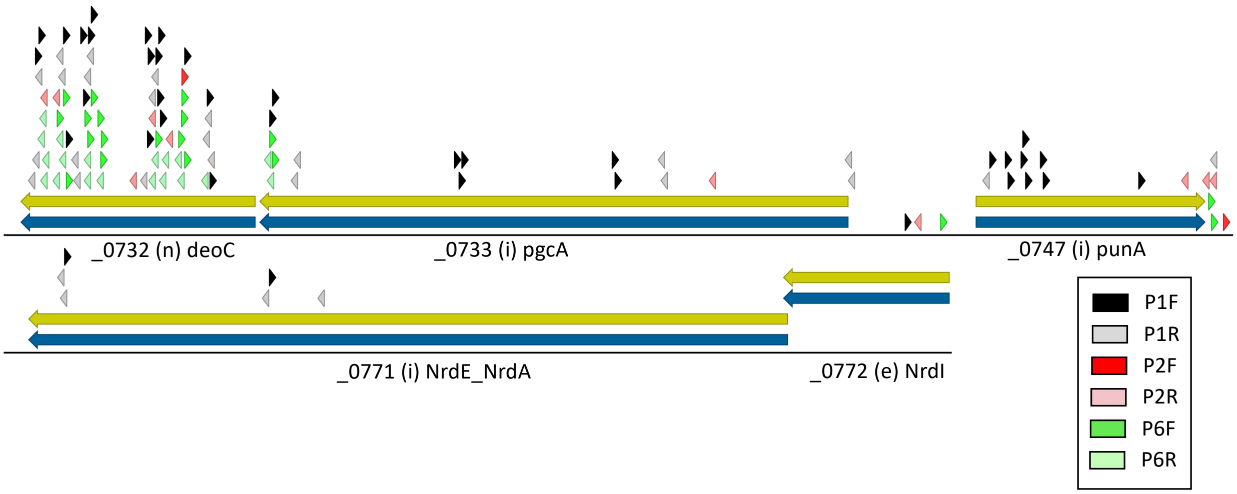

Fig. S4. **Gene _0747 shows an asymmetry in Tn*5*-Puro^R insertions but the reason is not obvious.**

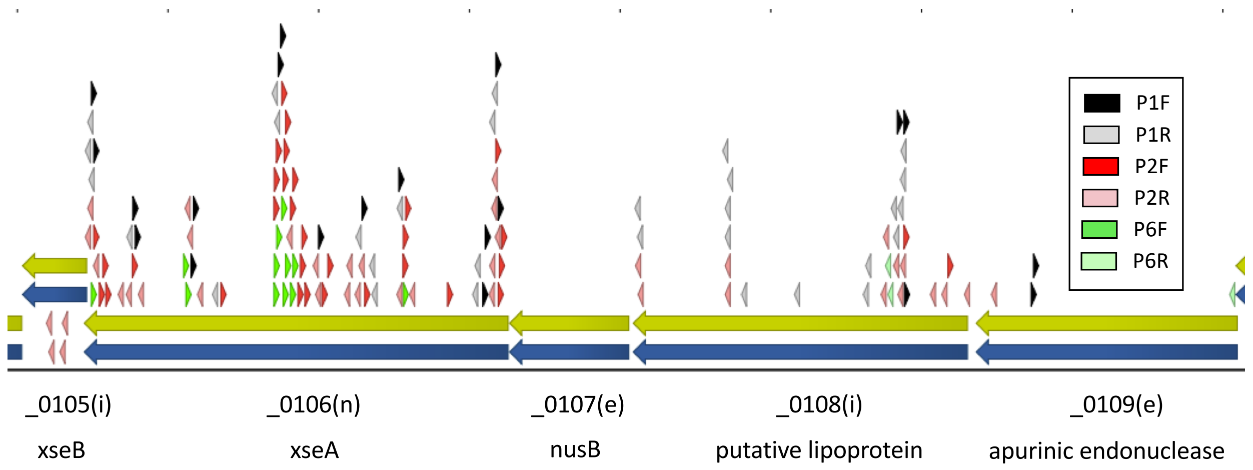

Figure S5. **Gene _0108 appears quasi-essential (i), apparently due to a polar effect on expression of the downstream essential (e) gene _0107.**

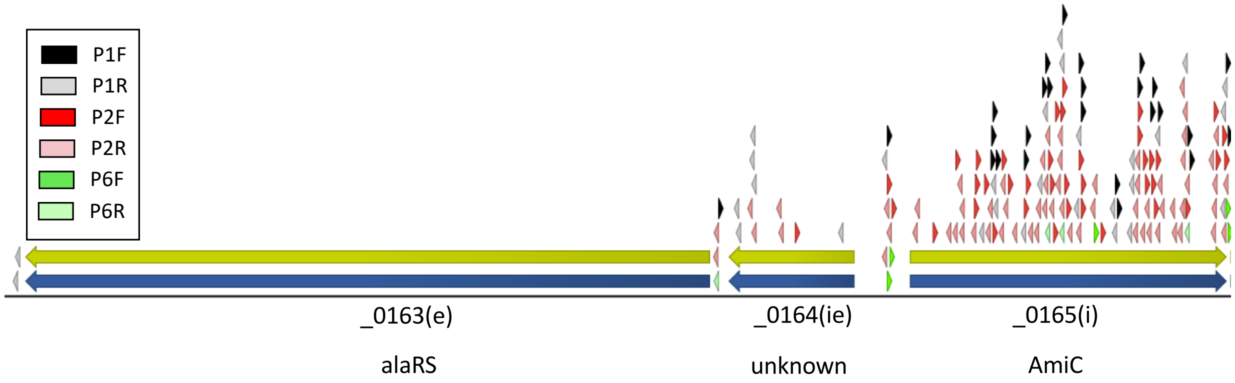

Figure S6. **Gene _0164 appears borderline between essential and quasi-essential (ie), apparently due to a polar effect on expression of the downstream essential (e) gene _0163.**

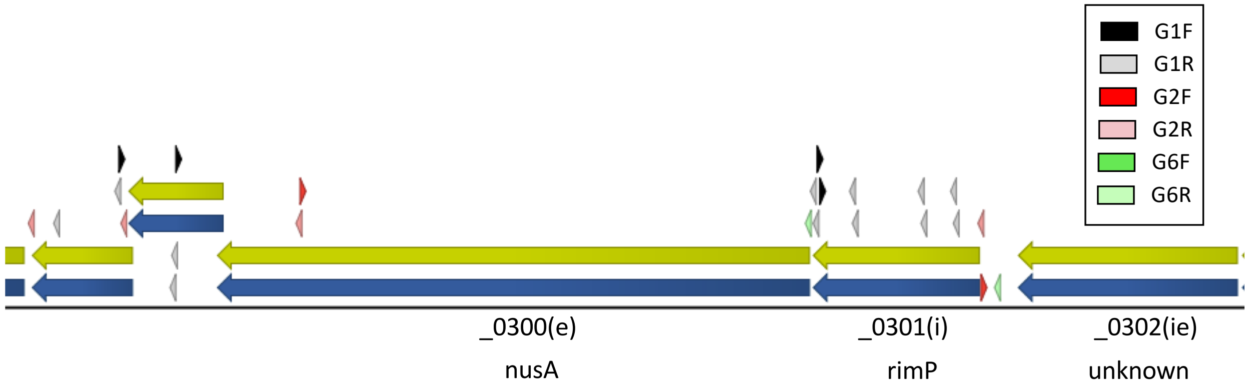

Figure S7. **Gene _301 appears quasi-essential (i), apparently due to a polar effect on expression of the downstream essential (e) gene _0300.**

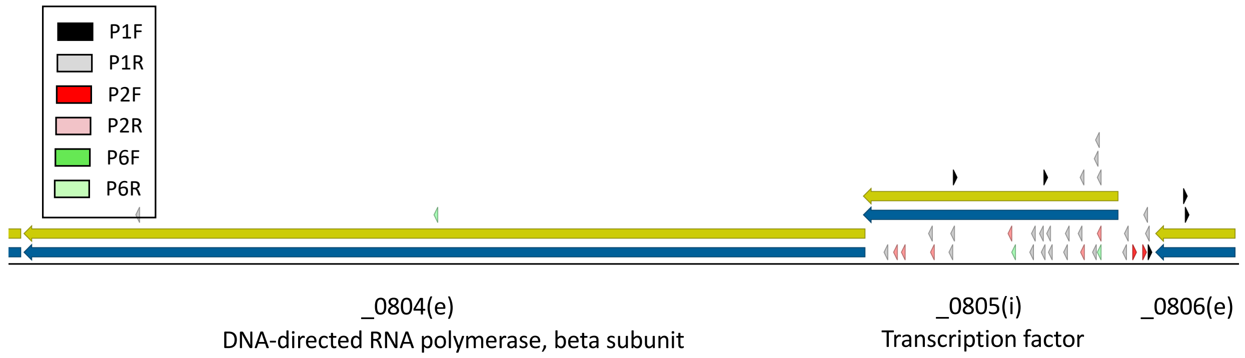

Figure S8. **Gene _0805 appears quasi-essential (i), apparently due to a polar effect on expression of the downstream essential (e) gene _0804.**

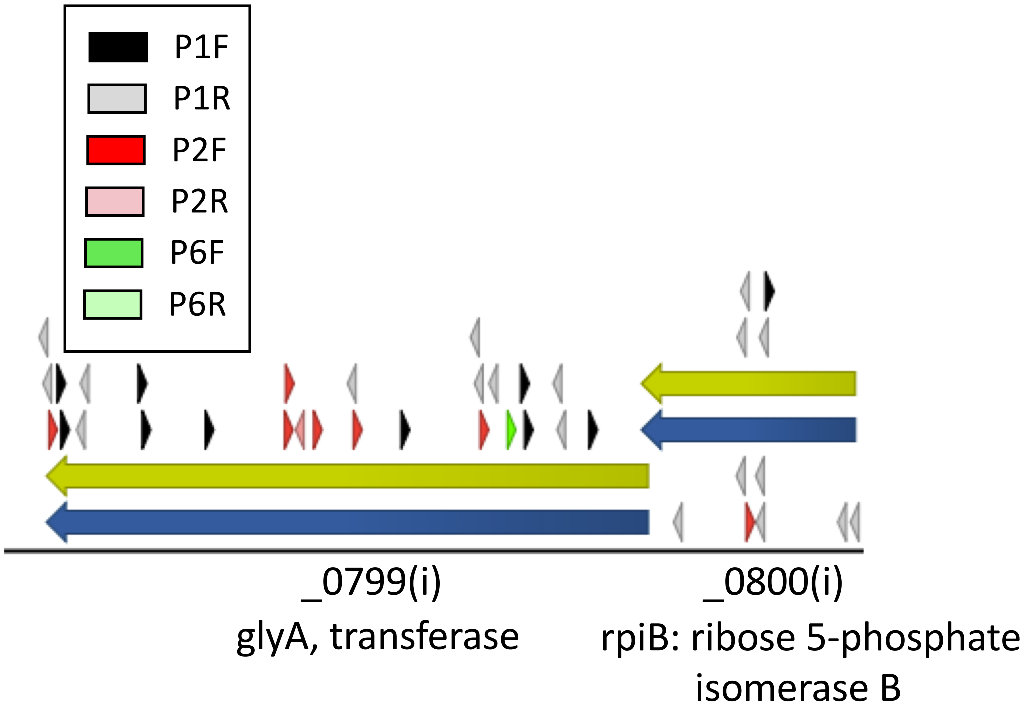

Figure S9. **Gene _0800 appears quasi-essential (i), apparently due to a polar effect on expression of the downstream essential (e) gene _0799.**

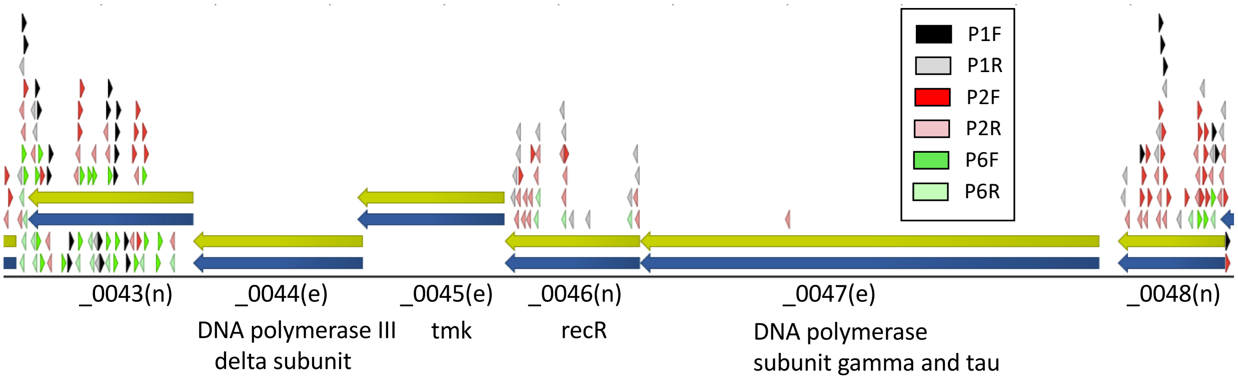

Figure S10. **Gene _0046 appears non-essential (n) and shows a skew in the orientation of Tn*5*-Puro[R] insertions, apparently due to a polar effect on expression of the downstream essential (e) gene _0045.**

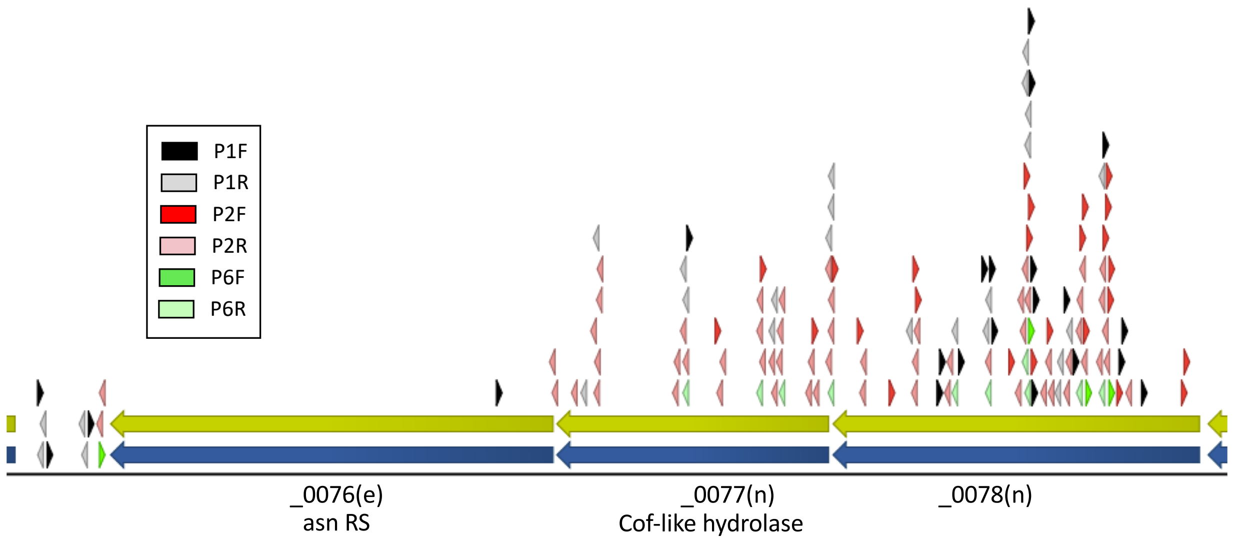

Figure S11. **Gene _0077 appears non-essential (n) and shows a skew in the orientation of Tn*5*-Puro^{R} insertions, apparently due to a polar effect on expression of the downstream essential (e) gene _0076.**

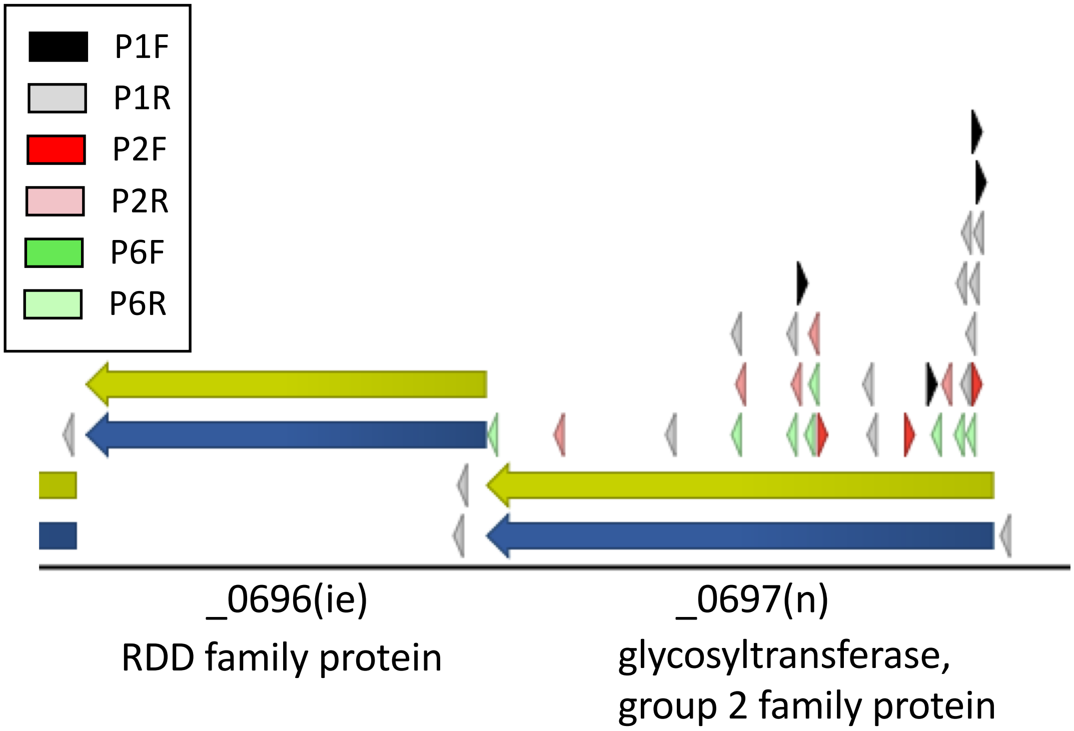

Figure S12. **Gene _0697 appears non-essential (n) and shows a skew in the orientation of Tn*5*-Puro^R insertions, apparently due to a polar effect on expression of the downstream gene _0697 (ie) on the borderline between quasi-essential and essential.**

Python script to identify forward and reverse Tn5-Puro^R insertion events

The code is presented in this .pdf file for your inspection. If you want to execute it then save it as an unformatted text file named “forward_reverse_xposon_fastq_v3.py”. The authors will supply the .py file upon request.

```
#!/usr/bin/python

#python forward_reverse_xposon_fastq_v3.py


#Setup.

import os

import sys

import time

vreadsz=25




#Files.

vq="C:\\"

vfile=os.listdir(vq)

vout0="C:\\out"

vgenome="CP002027.fasta"                    #CP002027.fasta, or CP016816.fasta

vseq="C:\\"+vgenome


#Get genome sequence

vA=[]

for a,b in enumerate(open(vseq)):

        b=b.strip()

        if(">" not in b):

                vA.append(b)

vtop="".join(vA)

vbot=vtop[::-1]

vbot=vbot.replace("A","X").replace("C","Y").replace("G","C").replace("T","A").replace("X","T").replace("Y","G")

vtopcircle=vtop+vtop[0:vreadsz-1]

vbotcircle=vbot+vbot[0:vreadsz-1]

vlong=len(vtop)


#Make hash of unique kmers (both strands, top and bottom).

vA={}

for a in range(0,vlong):

        if(vA.get(vtopcircle[a:a+vreadsz],"die")=="die"):

                vA[vtopcircle[a:a+vreadsz]]="t "+str(a)

        else:

                vA[vtopcircle[a:a+vreadsz]]=vA[vtopcircle[a:a+vreadsz]]+"\ttx "+str(a)

        if(vA.get(vbotcircle[a:a+vreadsz],"die")=="die"):
```

```
                    vA[vbotcircle[a:a+vreadsz]]="b "+str(a)
        else:
                    vA[vbotcircle[a:a+vreadsz]]=vA[vbotcircle[a:a+vreadsz]]+"\tbx "+str(a)
vktop={}
vkbot={}
vkdup={}
for a in vA:
        if("x" not in vA[a] and "t" in vA[a]):
                    vktop[a]="t"
        if("x" not in vA[a] and "b" in vA[a]):
                    vkbot[a]="b"
        if("x" in vA[a]):
                    vkdup[a]=vA[a]
print("\n\nGenomes parsed and kmers made. Start searching reads for transposon inserts.\nThis may take awhile...\n")


#Get reads
#GCCAACGACTACGCACTAGCCAACAAGAGCTTCA.GG.GTTG.AGATGTGTATAAGAGACAG (PURO cassette.19bp repeat)
#          TCATCGATGA.TG.GTTG.AGATGTGTATAAGAGACAG (PURO cassette.19bp repeat, bottom strand 5' to 3')
# vfm="GTTGAGATGTGTATAAGAGACAG"
vfm="GGGTTGAGATGTGTATAAGAGACAG"
vrm="TGGTTGAGATGTGTATAAGAGACAG"
look4sz=25
vsorted=[]
for c in vfile:
        if("fastq" not in c):
                    continue


        vname=c[0:c.find(".")]
        print(vname)
        vfound={}
        vdbl={}


        for a,b in enumerate(open(vq+c)):


                    #Skip QC lines in fastq files.
                    if((a+1)%2!=0 or (a+1)%4==0):
                                continue


                    #Process sequence lines in fastq files.
                    if((a+1)%2==0 and (a+1)%4!=0):
                                b=b.strip()


                                # Right end of xposon.
```

```
if(vfm in b):
    v1=b[b.find(vfm)+look4sz:]          #Read must have bit 'o marker seq joined to Tn5 19bp sequence (vfm).
    if(len(v1)>=vreadsz):               #Read must be long enough to be unique
        v2=v1[0:vreadsz]                #Grab the 30 bp or so to the right of Tn5 end.

        # Deal with reads of sequences that occur more than once in a genome.
        if(vkdup.get(v2,"die")!="die"):
            vloc=str(vtop.find(v2))     # This is the numerical location.
            if(vdbl.get(vloc,"die")=="die"):
                vdbl[vloc]="x"
            continue

        #Is the 30bp in the top strand? If so, record unique events.
        if(vktop.get(v2,"die")!="die"):     # Unique top strand sequence?
            vloc=str(vtop.find(v2))     # This is the numerical location.
            if(vfound.get(vloc,"die")=="die"):
                vfound[vloc]="RT"
            else:
                if("RT" not in vfound[vloc] and "LB" not in vfound[vloc]):
                    vfound[vloc]=vfound[vloc]+" RT"

        #Is the 30bp in the bot strand? If so, record unique events.
        if(vkbot.get(v2,"die")!="die"):
            vloc=str(vlong-(vbot.find(v2)))     # This is the numerical location.
            if(vfound.get(vloc,"die")=="die"):
                vfound[vloc]="RB"
            else:
                if("RB" not in vfound[vloc] and "LT" not in vfound[vloc]):
                    vfound[vloc]=vfound[vloc]+" RB"

# Left end of xposon.
if(vrm in b):
    v1=b[b.find(vrm)+look4sz:]          #Read must have bit 'o marker seq joined to Tn5 19bp sequence (vrm).
    if(len(v1)>=vreadsz):               #Read must be long enough to be unique
        v2=v1[0:vreadsz]                #Grab the 30 bp or so to the right of Tn5 end.

        # Deal with reads of sequences that occur more than once in a genome.
        if(vkdup.get(v2,"die")!="die"):
            vloc=str(vtop.find(v2))     # This is the numerical location.
            if(vdbl.get(vloc,"die")=="die"):
                vdbl[vloc]="x"
            continue
```

```
                #Is the 30bp in the top strand? If so, record unique events.
                if(vktop.get(v2,"die")!="die"):                    # Unique top strand sequence?
                    vloc=str(vtop.find(v2))                        # This is the numerical location.
                    if(vfound.get(vloc,"die")=="die"):
                        vfound[vloc]="LT"
                    else:
                        if("RB" not in vfound[vloc] and "LT" not in vfound[vloc]):
                            vfound[vloc]=vfound[vloc]+" LT"

                #Is the 30bp in the bot strand? If so, record unique events.
                if(vkbot.get(v2,"die")!="die"):
                    vloc=str(vlong-(vbot.find(v2)))                # This is the numerical location.
                    if(vfound.get(vloc,"die")=="die"):
                        vfound[vloc]="LB"
                    else:
                        if("RT" not in vfound[vloc] and "LB" not in vfound[vloc]):
                            vfound[vloc]=vfound[vloc]+" LB"

    for a in vfound:
        if("RT" in vfound[a] or "LB" in vfound[a]):
            vsorted.append(a.zfill(7)+" "+vname+" fwd\n")
        if("RB" in vfound[a] or "LT" in vfound[a]):
            vsorted.append(a.zfill(7)+" "+vname+" rev\n")


# Write file. To import into clc
#    source    1..1078809
vf1=open(vout0,'w')
for c,d in enumerate(vsorted):
    d=d.rstrip()
    v1=d.split(" ")
    if(v1[2]=="fwd"):
        vf1.write((v1[1]+"F").center(21)+str(int(v1[0]))+".."+str(int(v1[0])+19)+"\n")
    if(v1[2]=="rev"):
        vf1.write((v1[1]+"R").center(21)+"complement("+str(int(v1[0])-19)+".."+str(int(v1[0]))+")\n")
vf1.close
```
